# Assessment of lung cancer risk factors and mortality in Qatar: A case series study

**DOI:** 10.1002/cnr2.1302

**Published:** 2020-10-07

**Authors:** Abdel‐Salam G. Abdel‐Salam, Mohammad Mollazehi, Dipankar Bandyopadhyay, Ahmed M. Malki, Zumin Shi, Hatem Zayed

**Affiliations:** ^1^ Department of Mathematics, Statistics and Physics College of Arts and Sciences, Qatar University Doha Qatar; ^2^ Department of Biostatistics, School of Medicine Virginia Commonwealth University Richmond Virginia USA; ^3^ Department of Biomedical Sciences College of Health Sciences, QU Health, Qatar University Doha Qatar; ^4^ Human Nutrition Department College of Health Sciences, QU Health, Qatar University Doha Qatar

**Keywords:** cox regression, Kaplan‐Meier curves, lung cancer, survival rate

## Abstract

**Background:**

The global burden of cancer has exponentially increased over the last few years. In 2018 alone, approximately more than half of the 18.1 million individuals who had cancer succumbed to it. Lung cancer cases and fatalities are particularly on the rise. Therefore, exploring the factors surrounding lung cancer mortality is of utmost importance.

**Aims:**

We investigate the clinicopathological and epidemiological characteristics of patients with lung cancer undergoing treatments, and their 5‐year survival rates from a case series study in Qatar.

**Methods and Results:**

All patients' data (between January 2010 and December 2014) in this case series study were retrieved from Al‐Amal Hospital database. Kaplan‐Meier survival plots, life tables and Cox regression were utilized for the statistical analysis. A total of 229 lung cancer patients were included in this study; of which 23.6% are Qatari (40 males and 14 females) and 76.4% non‐Qatari (133 males and 42 females). Approximately 57.6% of our patients received at least one type of treatment. We observe a 5‐year survival rate of 9.4% in our patient cohort. We also observe other predictive factors, such as distant metastasis (adjusted hazards ratio, HR = 2.414, 95% CI: 1.035‐5.632), smoking status (adjusted HR = 3.909, 95% CI: 1.664‐9.180) and the treatment history (adjusted HR = 0.432, 95% CI: 0.270‐0.691), to be significant.

**Conclusion:**

Lung cancer is a prevalent health condition in Qatar, particularly owing to the rising use of tobacco in the country. The survival rate for lung cancer patients in this country is lower, compared to the global average. Moreover, several factors such as distant metastasis, smoking status, and treatment history are associated with lung cancer survival in Qatar.

## 1 INTRODUCTION

The global burden of cancer is expeditiously increasing, contributing to significant economic problems in all countries.^1^ According to the International Agency for Research on Cancer, the global burden of cancer using the GLOBOCAN estimates was projected to be 18.1 million new cases in 2018, with 9.6 million dying from it.^2^ In particular, one in eight men and one in 11 women will be dying from the disease, with a 5‐year estimated prevalence (worldwide) to be 43.8 million. Lung cancer, often attributed to smoking and weak tobacco control laws, continues to remain one of the most commonly diagnosed cancer types in the world, and a leading cause of mortality, to the extent that it claims more lives compared to colon, prostate, ovarian, and breast cancers combined.^3^, ^4^ In 2018, there was a total of 2.1 million new lung cancer cases worldwide.^2^, ^5^ This emphasizes the importance of this malignancy in oncological studies. The Middle East Cancer Consortium performed a comprehensive epidemiological study on the prevalence of cancer in the Middle East region and observed that the incidences were lower than what was seen in the USA and Europe.^6^, ^7^

Primary lung cancer begins in the lungs and is unrelated to any previous cancer. Secondary lung cancer can be of various types, such as a second and unrelated cancer (called the second primary) in someone with prior lung cancer, lung metastases from cancer of any other region (such as the breast), or spreading from the lung to other areas. Primary lung cancer is generally of two types, small cell, and non‐small cell. Smoking is the leading cause of most small cell lung cancer (SCLC) cases, and makes up approximately 12% of diagnosed lung cancers. The remaining 88% is non‐small cell lung cancer (NSCLC).^6^, ^8^ Continued exposure to smoke particles that are full of carcinogens damages the cells that line the lung, leading them to act abnormally and causing cancer.^9^ This includes both active and passive smokers. Other causes of lung cancer include exposure to carcinogenic agents, such as radon or inhaling toxic chemicals, such as arsenic, cadmium, chromium, nickel, uranium, and other petroleum chemicals.^10^ Other important risk factors include a family history of lung cancer, and any previous radiation exposure, that could have been subjected to the chest.^8^

One of the standard treatment options for lung cancer is surgery, primarily if the tumor is still localized and not metastasized. A surgical operation can include the removal of the primary cancerous tissue from the lung and some from the neighboring healthy tissue. This is known as a wedge resection. Whereas surgical intervention that includes the removal of a large part of the lung is called segmental resection.^11^

Factors affecting life expectancy with lung cancer include age, sex, race, lifestyle, and preexisting conditions. Life expectancy has been increasing alongside innovations in cancer treatment. In the 1970s, it was estimated that about 12.5% of the patients would live beyond 5 years of age with lung cancer. This increased to 17.3% in 2010 and varied depending on the type of cancer.^12^ Patients diagnosed with cancer at the early stages, like stage one and two, have a longer life expectancy than those diagnosed at stage three or four.

Qatar's healthcare system exhibits average performance regarding the preparedness and response to cancer care.^13^ Qatar's mixed healthcare delivery system includes both the public and the private sector, which provide primary and secondary cancer care, for instance, surgery radiotherapy and chemotherapy services. In the Middle East region, Qatar has amongst the highest healthcare budget to cater to both the natives and non‐Qataris. Cancer care is available at a standardized annual fee where the nationals pay US$3 under the established government leading healthcare provider Hamad Medical Corporation. The non‐nationals pay US$28 fee yearly, with the government requiring each employer to provide health coverage for the non‐residents.^13^ Qatar experiences population growth annually due to expatriates accounting for 94 different nationalities as of the year 2019, with India as the dominant, and Bangladesh, Nepal, Egypt, and the Philippines among the top five nationalities.^14^ In 2015, only the newly detected cases of cancer were 1466, with 18% and 82% distributed among the Qataris and Non‐Qataris, respectively.^15^ However, with a simplistic healthcare system, Qatar still experiences problems as it does not warrant a more comprehensive initiative for cancer treatment, and has also not invested significantly in research and stringent prevention measures.

In Qatar, the prevalence of all types of cancer is low, with 175 recorded mortalities in 2008.^12^ In line with the 2015 yearly report of the Qatar National Cancer Registry, more than 16.46% of cancer mortalities amongst its population resulted from lung cancer, with approximately 90% of the entire lung cancer cases linked to tobacco use.^15^ In 2018, according to the World Health Organization, lung cancer was responsible for the death of 68 people in Qatar, which was about 10.1% of the total recorded mortalities due to cancer.^16^ Moreover, the age‐standardized incidence rate of lung cancer in Qatar between 1998 to 2005 was estimated to be 8.95/100 000/year (15.2/100 000 for males; 3.95/100 000 for females). Compared to other Gulf Cooperation Council (GCC) Countries, these rates were higher.^17^

The epidemiological characteristics of lung cancer and their associated risks have not been thoroughly investigated within the kingdom of Qatar. Hence, the objectives of this study are: (a) to investigate the epidemiological characteristics of lung cancer and associated risk, and (b) to examine the 5‐year survival rate of lung cancer in Qatar, from a case series study of lung cancer patients recruited between January 2010 and December 2014.

## 2 METHODS

The current study is a retrospective study of the demographic and epidemiological characteristics of lung cancer patients in Qatar. Data has been obtained from the research database of the National Center for Cancer Care and Research (Al‐Amal Hospital). Patients included in this study were diagnosed with primary lung cancer from January 2010 to December 2014 (inclusive). The total number of lung cancer patients registered in the given period in Al‐Amal Hospital was 229 patients.

For analysis, the statistical package for social science (SPSS)^18^ and *R*‐Studio^19^ were used. Pearson Chi‐square test was used to assess the association between treatment history and various patient characteristics. This test provides information on the significance of any observed differences, as well as on the specific groups or categories which are responsible for the differences observed.^20^ Kaplan‐Meier curves^21^ were plotted to estimate survival probability, separately for the patients' characteristics, such as gender, nationality, cancer types, etc. The life table was used to determine the *k*th‐year survival rate, *k* = 1, …, 5. Finally, Cox proportional hazards (PH) regression^22^ was used to quantify the relationship between the surviving time of lung cancer patients and the patient epidemiological/biological characteristics. We also investigated the adequacy of the PH regression via the Schoenfeld test.^23^ In a nutshell, use of the Cox regression model allows the researcher to scientifically conduct an analysis of the prognosis and survival rate of individuals diagnosed with carcinoma of the lung, as well as enabling the identification of the various variables which impact on their survival.

## 3 RESULTS

### 3.1 Patients' characteristics

The demographic and clinical characteristics of the 229 lung cancer patients are presented in Table 1. Out of these, 75.5% were men and 24.5% were women. With respect to age groups, 25.8% of patients were aged below 50, 33.6% of patients were aged between 50 and 59 years, 21.8% of patients were between 60 and 99 years whereas 18.8% of patients were 70 years or more. Qatari citizens accounted for 23.6% of patients, while 76.4% were non‐Qataris. The percentage of patients diagnosed with NSCLC was 78.2%, while the remaining were diagnosed with SCLC (21.8%). Approximately 85.6% of the patients had distant metastasis, while 14.4% did not have. Out of all these patients, 57.6% had had at least one type of treatment (ie, surgery, radiotherapy or chemotherapy).

**TABLE 1:** Demographical and clinical characteristics of lung cancer patients in Qatar between 2010 and 2014

	N	%
Gender		
Women	56	24.5
Men	173	75.5
Age groups		
Less than 50	59	25.8
50‐59	77	33.6
60‐69	50	21.8
70+	43	18.8
Nationality		
Non‐Qatari	175	76.4
Qatari	54	23.6
Cancer type		
SCLC	50	21.8
NSCLC	179	78.2
Distant metastasis		
No	33	14.4
Yes	196	85.6
Treatment		
No	97	42.4
Yes	132	57.6
Smoking status at the time of cancer diagnosis		
Non‐smoker	43	18.8
Smoker	186	81.2
Vital status by December 2014		
Alive	133	58.1
Dead	96	41.9

The majority of patients (81.2%) were smokers at the time of diagnosis. Among the total cases of patients included in this study, 41.9% experienced the event of interest by the end of December 2014 (ie, death from lung cancer).

Table 2 shows various patient characteristics varying with treatment variables. We observe that the percentage of women who underwent treatment was 58.9%. On the other hand, the percentage of men who were treated was 57.2%. With respect to age groups, 66.1% of patients aged below 50 years, 50.6% of patients aged between 50 and 59 years, 66% of patients aged between 60 and 99 years, and 48.8% of patients aged 70 years or more underwent treatment. Considering patient nationality, the percentage of Qataris who underwent treatment was 55.6%. These low percentages might be due to the foreign medical treatment program offered by the government. On the other hand, the percentage of non‐Qataris who sought medical intervention was 58.3%. Among patients with SCLC, 38% were attended to; this represented 63.1% of patients with NSCLC. Furthermore, this data shows that among the patients having distant metastasis, 55.6% were offered therapy. On the contrary, among patients who did not have distant metastasis, 69.7% received medical attention. Additionally, the percentage of patients who were smokers at the time of diagnosis and were receiving treatment was 57.2%. On the other hand, the percentage of patients who were not smokers at the time of diagnosis and who had been put on therapy was 72.9%. Finally, the vital status report provided in December 2014 showed that the percentage of patients who survived after receiving treatment was 72.9%. Moreover, the percentage of patients who died after receiving treatment was 36.5%. Table S1 shows patient characteristics varying with vital status.

**TABLE 2:** Patient characteristics across the treatment history

	Treatment		
	N	%	*P*‐value
Gender			
Women	33	58.9	.823
Men	99	57.2	
Age group			
Less than 50	39	66.1	.108
50‐59	39	50.6	
60‐69	33	66	
70+	21	48.8	
Nationality			
Non‐Qatari	102	58.3	.723
Qatari	30	55.6	
Cancer type			
SCLC	19	38	.001
NSCLC	113	63.1	
Distant metastasis			
No	24	72.7	.058
Yes	108	55.1	
Smoking status at the time of cancer diagnosis			
Non‐Smoker	34	79.1	.002
Smoker	98	57.2	
Vital status by December 2014			
Alive	97	72.9	<.001
Dead	35	36.5	

*Note*: The *P*‐values correspond to the chi‐square tests.

### 3.2 Log‐rank tests for evaluating group differences

**FIGURE 1:**
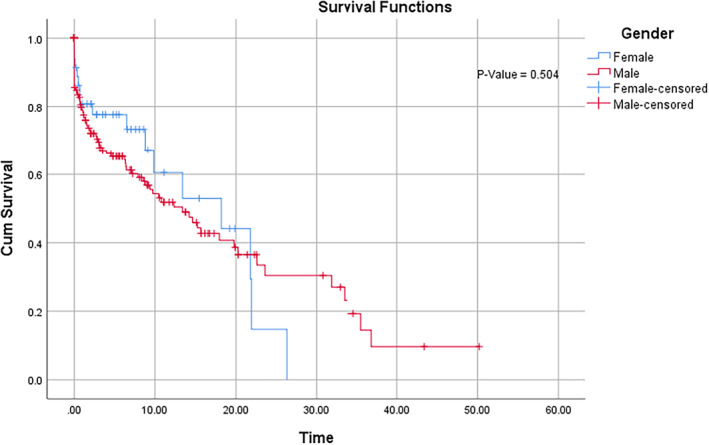
Kaplan–Meier survival curves for lung cancer patients varying with gender

Table 3 presents the median survival times for the various subject characteristics and the corresponding *P*‐values (obtained from log‐rank tests) that depicts the difference between the different groups. For example, the median survival time for female and male are 18.2 and 13.4 months, respectively. However, the log‐rank test revealed that this difference is not statistically significant. Figure 1 also shows that the survival curves for both groups do not separate significantly from each other. This is consistent with the log‐rank test result. From the figure, it clearly shows a lower survival in men in the first 20 months.

**TABLE 3:** Median survival time (months) of lung cancer patients by sociodemographic characteristics, cancer types, and treatment history

Characteristics	Median	*P*‐value
Gender		
Women	18.20	.504
Men	13.40	
Nationality		
Non‐Qatari	15.27	.048
Qatari	3.53	
Type		
SCLC	12.37	.041
NSCLC	17.97	
Distant metastasis		
No	15.67	.006
Yes	10.47	
Smoking Status		
Non‐smoker	35.53	<.001
Smoker	9.83	
Treatment		
Yes	21.93	<.001
No	3.13	

Moreover, the median survival time for Qatari and non‐Qatari are 3.53 and 15.57 months, respectively. The log‐rank test showed that this difference is statistically significant (*P*‐value = .048); see Figure S1. Comparing cancer types, the median survival time for patients with SCLC and NSCLC were 12.37 and 17.97 months, respectively. This difference is significant (*P*‐value = .041) based on the log‐rank test; see Figure S2. The median survival time (10.47 months) for patients with distant metastasis was significantly shorter than patients with no distant metastasis (15.67 months); Kaplan‐Meir plots and log‐rank based *P*‐value is in Figure S3. Moreover, the median survival time of smokers (8.83 months) was substantially different from non‐smokers (35.53 months). Finally, in terms of treatment, there was a statistically significant difference between patients who did not have any treatment, compared to those who had been offered at least one type of cancer therapy. The Kaplan‐Meier plots and log‐rank based *P*‐values the smokers/non‐smokers and treatment/no treatment groups appear in Figures S4 and S5.

### 3.3 Stratified Cox proportional hazards regression

To investigate the effect of plausible demographics, socio‐behavioral, and treatment characteristics on lung cancer survival, we use the stratified Cox proportional hazard (PH) regression, stratified by age group. However, prior to model fitting, we evaluate the PH assumption for each covariate via the Schoenfeld test.^23^ Table 4 presents the results from the corresponding Chi‐squared test, and the two‐sided *P*‐values. Furthermore, Figure S6 plots, for each covariate, the scaled Schoenfeld residual, against the transformed time. From the table, we observe that none of the tests, including the global test, is significant at the 5% level. Hence, we can conclude that the underlying PH assumptions are valid, and we can proceed with the stratified Cox PH fit.

**TABLE 4:** Chi‐Square test statistics, degrees of freedom (*df*) and corresponding *P*‐values from the Schoenfeld test for evaluating proportional hazards assumption

Factors	Chi‐Square	*df*	*P*‐value
Gender	0.899	1	.343
Nationality	0.077	1	.781
Type	0.155	1	.694
Distant metastasis	3.126	1	.077
Smoking status	0.395	1	.530
Treatment	1.746	1	.186
Global test^a^	8.362	6	.213

^a^ The global test is based on stratified Cox model.

Results from the stratified Cox PH fit are summarized in Table 5, where covariate effects can be explained in terms of hazard ratio (HR) and the corresponding 95% confidence interval (CI). We observe significance with regards to distant metastasis, treatment, and smoking status.

**TABLE 5:** Adjusted Hazard ratio and 95% confidence intervals for all‐cause mortality among lung cancer patients (N = 229)

Factors	Adjusted HR^a^ ^,^ ^b^	95% CI HR		*P‐*value
		Lower	Upper	
Gender				
Women	0.623	0.349	1.110	.108
Men	Ref			
Nationality				
Non‐Qatari	0.669	0.405	1.107	.118
Qatari	Ref			
Cancer type				
SCLC	1.144	0.715	1.831	.575
NSCLC	Ref			
Distant metastasis				
Yes	2.414	1.035	5.632	.041
No	Ref			
Treatment				
Yes	0.432	0.270	0.691	<.001
No	Ref			
Smoking Status				
Smoker	3.909	1.664	9.180	.002
Non‐Smoker	Ref			

^a^ Model adjusted for gender, nationality, cancer type, distant metastasis, treatment, and smoking status.

^b^ Model is stratified by age groups.

Abbreviation: HR, hazard ratio.

The risk of death for patients with distant metastasis cancer is 141.4% higher (HR = 2.414, 95% CI: 1.035, 5.632), compared to other patients. Similarly, the hazard of death for subjects in the treatment group is 56.8% lower (HR = 0.432, 95% CI: 0.266, 0.681) compared to the group without treatment. Finally, the risk of death for smoker patients is 290.9% higher (HR = 3.909, 95% CI: 1.664, 9.180) compared to non‐smokers.

### 3.4 Life table

For evaluating yearly survival rates of lung cancer patients, a life table (Table 6) was also constructed. This table presents the number of censored and failed patients, the corresponding survival rate, and the SE, for the first 5 years, allowing us to understand and evaluate these quantities on a yearly basis. From the table, the 5‐year survival rate of lung cancer patients is 9.4%. The corresponding life tables for men and women patients are in the Tables S2 and S3. While the 5‐year survival rate for males is 0.106, the corresponding rate for the females is higher, 0.263.

**TABLE 6:** Life table for survival estimates of lung cancer patients (N = 229)

Years	Interval (Month)		Failed	Censored	Survival rate	SE
	Lower	Upper				
first	0	12	75	109	1.000	0.000
second	12	24	15	19	0.570	0.038
third	24	36	5	3	0.329	0.052
fourth	36	48	1	1	0.156	0.059
fifth	48	60	0	1	0.094	0.060

Abbreviation: SE, standard errors.

## 4 DISCUSSION

Lung cancer is a prevalent health condition in Qatar, owing primarily to the rising tobacco utilization in the country. In light of the global (average) 5‐year survival rate of 15%,^24^ the survival rate for lung cancer patients in Qatar is lower, about 9.4%. The corresponding 5‐year survival rate in counties, such as the United States, the United Kingdom, Saudi Arabia, and Libya, are respectively, 18.6%, 14.7%, 14%, and 2%.^25^ Aside from Libya, Qatar ranks lower, providing a basis for improving lung cancer survival rates.

Various factors are related to lung cancer survival in Qatar, such as age, distant metastasis, smoking status, and chemotherapy. Various other studies^26^, ^27^ corroborate our findings. For example, elderly patients (≥65 years) have increased hazards of developing lung cancer, compared to the younger.^26^ Additional factors recognized in the literature are smoking duration, family history of lung cancer, histology, and history of infarction/heart disease.^27^ The high prevalence of smoking among patients is in line with the current knowledge of the link between smoking and lung cancer. In Qatar, the prevalence of smoking was 16.4% (31.9% in men and 1.2% in women).^28^ Strict smoking regulations should be in place to prevent lung cancer and other health problems.

## 5 CONCLUSION

Our analysis of the Qatari lung cancer population between 2010 and 2014 provides us with an avenue for understanding the primary factors influencing the time to event of lung cancer. Evaluating the 5‐year survival rate was critical, in that it offers sufficient allowance for studying the progress of the condition, and hence determining whether Qatar has realized any progress in dealing with the disease. However, a fundamental limitation attributed to this study is the absence of other pertinent sociodemographic information, such as employment status, income level, and education. This information would have assisted us in understanding whether specific groups have higher chances of lung cancer survival, compared to others.^29^

Recent research^30^ reveals that with increasing age, the risk of surgery increases, with deaths due to noncancer‐specific causes becoming more prominent. These noncancer‐specific deaths can be attributed to “competing risk” events,^31^ a topic of considerable interest and relevance for clinicians, given that a vast majority of lung cancer patients are elderly with a median age at diagnosis of 70 years, and effective treatment plans are necessary to target a known disease (lung cancer), in the face of unavoidable risk of death due to old age. However, our current database does not have information on competing risks of lung cancer, and we plan to pursue this in the future.

Our current effort led to the creation of a reliable, accessible, and accurate database combining various medical records on lung cancer cases in Qatar. Considering this as the stepping stone, we expect future research to focus on integrating a comprehensible list of risk factors, thereby allowing us to refine our findings, and thus provide a significantly more precise picture of lung cancer survival in Qatar. The findings derived can lead to steps that the country can adopt to improve the situation. We must also highlight the industrialization of Qatar, and the associated environmental risks will also affect future studies on lung cancer cases. This will also be an essential area for future research, based on potential occupational exposure as a variable.

## ETHICAL STATEMENT

Ethical Approval for this secondary retrospective analysis was obtained by the Institutional Review of Board of QU.

## CONFLICT OF INTEREST

The authors declared no potential conflicts of interest with respect to the research, authorship, and/or publication of this article.

## AUTHOR CONTRIBUTIONS

All authors had full access to the data in the study and take responsibility for the integrity of the data and the accuracy of the data analysis. *Conceptualization*, A.‐S. G. A.‐S., D. B.; *Formal Analysis*, A.‐S. G. A.‐S., M. M., D. B.; *Methodology*, A.‐S. G. A.‐S., M. M., D. B.; *Project Administration*, A.‐S. G. A.‐S.; *Validation*, A.‐S. G. A.‐S., M. M., D. B.; *Visualization*, A.‐S. G. A.‐S., M. M., D. B.; *Writing Original Draft*, A.‐S. G. A.‐S., M. M.; *Writing Review Editing*, A.‐S. G. A.‐S., M. M., D. B.; *Data Curation*, M. M.; *Investigation*, M. M., D. B.; *Resources*, M. M.; *Funding Acquisition*, D. B.

## Supporting information


**Data S1.** Supporting Information.

## Data Availability

The data is available upon request.

## References

[1] Fitzmaurice C , Dicker D , Pain A , et al. The global burden of cancer 2013. JAMA Oncol. 2015;1(4):505‐527. 26181261 10.1001/jamaoncol.2015.0735 PMC4500822

[2] Bray F , Ferlay J , Soerjomataram I , Siegel RL , Torre LA , Jemal A . Global cancer statistics 2018: GLOBOCAN estimates of incidence and mortality worldwide for 36 cancers in 185 countries. CA Cancer J Clin. 2018;68(6):394‐424. 30207593 10.3322/caac.21492

[3] World Health Organization Factsheet . [cited 2020Aug23]. Retrieved from https://www.who.int/news-room/fact-sheets/detail/cancer

[4] Islami F , Torre L , Jemal A . Global trends of lung cancer mortality and smoking prevalence. Transl Lung Cancer Res. 2015Aug;4(4):327‐338. 26380174 10.3978/j.issn.2218-6751.2015.08.04 PMC4549470

[5] Shankar A , Saini D , Dubey A , et al. Feasibility of lung cancer screening in developing countries: challenges, opportunities and way forward. Transl Lung Cancer Res. 2019;8(S1):106‐121. 10.21037/tlcr.2019.03.03 PMC6546626 31211111

[6] Torre LA , Bray F , Siegel RL , Ferlay J , Lortet‐Tieulent J , Jemal A . Global cancer statistics, 2012. CA Cancer J Clin. 2015;65(2):87‐108. 25651787 10.3322/caac.21262

[7] Ferlay J , Shin H‐R , Bray F , Forman D , Mathers C , Parkin DM . Estimates of worldwide burden of cancer in 2008: GLOBOCAN 2008. Int J Cancer. 2010;127(12):2893‐2917. 21351269 10.1002/ijc.25516

[8] Molina JR , Yang P , Cassivi SD , Schild SE , Adjei AA . Non‐small cell lung cancer: epidemiology, risk factors, treatment, and survivorship. Mayo Clin Proc. 2008;83(5):584‐594. 18452692 10.4065/83.5.584 PMC2718421

[9] Field RW , Withers BL . Occupational and environmental causes of lung cancer. Clin Chest Med. 2012;33(4):681‐703. 23153609 10.1016/j.ccm.2012.07.001 PMC3875302

[10] Tchounwou PB , Yedjou CG , Patlolla AK , Sutton DJ . Heavy metal toxicity and the environment. In: Luch A. (ed.). Molecular, Clinical and Environmental Toxicology. Experientia Supplementum, vol 101. Springer, Basel; 2012. 10.1007/978-3-7643-8340-4. PMC4144270 22945569

[11] Davis JN , Medbery C , Sharma S , et al. Stereotactic body radiotherapy for centrally located early‐stage non‐small cell lung cancer or lung metastases from the RSSearch® patient registry. Radiat Oncol. 2015;10(1):113. 25975848 10.1186/s13014-015-0417-5 PMC4443630

[12] Habib Toumi BBC . Cancer the third cause of deaths in Qatar [internet]. Gulf News; 2018 [cited 2020Aug23]. Retrieved from https://gulfnews.com/world/gulf/qatar/cancer-the-third-cause-of-deaths-in-qatar-1.758721

[13] Howitt PJ , Kerr K , Kuwari HA , Ali FMH , Knuth A , Darzi A . Challenges in adapting international best practices in cancer prevention, care, and research for Qatar. Health Aff. 2014;33(9):1635‐1640. 10.1377/hlthaff.2014.0381 25201669

[14] Snoj J . Population of Qatar by nationality in 2019 [Internet]. 2019 [cited 2020Aug23]. Retrieved from: https://priyadsouza.com/population-of-qatar-by-nationality-in-2017/

[15] Qatar Cancer Incidence Report , 2015 [Internet]. Qatar National Cancer Registry. Ministry of Public Health; 2017 [cited 2020Aug23]. Retrieved from: https://www.moph.gov.qa/_layouts/15/download.aspx?SourceUrl=/Admin/Lists/PublicationsAttachments/Attachments/53/QNCR-2015-English.pdf

[16] Qatar factsheet [Internet] . World Health Organization ‐ Cancer Country Profile. 2020 [cited 2020Aug23]. Retrieved from https://www.who.int/cancer/country-profiles/QAT.PDF?ua=1

[17] Ibrahim WH , Rasul KI , Khinji A , Ahmed MS , Bener A . Clinical and epidemiological characteristics of lung cancer cases in Qatar. EMHJ‐East Mediterr Health J. 2010;16(2):166‐170. 20799569

[18] IBM Corp. Released . IBM SPSS Statistics for Windows, Version 26.0. Armonk, NY: IBM Corp; 2019.

[19] RStudio Team . RStudio: Integrated development environment for R. Boston, MA: RStudio, PBC; 2020.

[20] Akins CW , Miller DC , Turina MI , et al. Guidelines for reporting mortality and morbidity after cardiac valve interventions⋆. Eur J Cardiothorac Surg. 2008;33(4):523‐528. 18313319 10.1016/j.ejcts.2007.12.055

[21] Rich JT , Neely JG , Paniello RC , Voelker CCJ , Nussenbaum B , Wang EW . A practical guide to understanding Kaplan‐Meier curves. Otolaryngol Head Neck Surg. 2010;143(3):331‐336. 20723767 10.1016/j.otohns.2010.05.007 PMC3932959

[22] Bellera CA , Macgrogan G , Debled M , de Lara CT , Brouste V , Mathoulin‐Pélissier S . Variables with time‐varying effects and the cox model: some statistical concepts illustrated with a prognostic factor study in breast cancer. BMC Med Res Methodol. [Epub 2010 Mar 16. PMID: 20233435; PMCID: PMC2846954]. 2010;10:20. 10.1186/1471-2288-10-20. 20233435 PMC2846954

[23] Schoenfeld D . Chi‐squared goodness‐of‐fit tests for the proportional hazards regression model. Biometrika. 1980;67(1):145‐153.

[24] Yang P . Epidemiology of lung cancer prognosis: quantity and quality of life. Methods Mol Biol Cancer Epidemiol. 2009;471:469‐486. 10.1007/978-1-59745-416-2_24 PMC2941142 19109795

[25] Cheng T‐YD , Cramb SM , Baade PD , Youlden DR , Nwogu C , Reid ME . The international epidemiology of lung cancer: latest trends, disparities, and tumor characteristics. J Thorac Oncol. 2016;11(10):1653‐1671. 27364315 10.1016/j.jtho.2016.05.021 PMC5512876

[26] Alghamdi HI , Alshehri AF , Farhat GN . An overview of mortality & predictors of small‐cell and non‐small cell lung cancer among Saudi patients. J Epidemiol Global Health. [Epub 2017 Sep 28. PMID: 29801587; PMCID: PMC7386448]. 2018;7(S1):S1‐S6. 10.1016/j.jegh.2017.09.004. PMC7386448 29801587

[27] Warkentin MT , Tammemägi MC , Freedman MT , et al. Factors associated with small aggressive non–small cell lung cancers in the National Lung Screening Trial: a validation study. JNCI Cancer Spectrum. 2018;2(1):1‐10. 10.1093/jncics/pkx010 PMC6649725 31360836

[28] Tobacco Cessation [Internet]. Ministry of Public Health; [cited 2020Aug23]. Retrieved from https://phs.moph.gov.qa/data/smoking/

[29] Wong MCS , Lao XQ , Ho K‐F , Goggins WB , Tse SLA . Incidence and mortality of lung cancer: global trends and association with socioeconomic status. Sci Rep. 2017;7(1). 10.1038/s41598-017-14513-7 PMC5662733 29085026

[30] Kim JY . Analyzing competing risks in the treatment of lung cancer: a good start. J Thorac Dis. 2017;9(3):474‐476. 28449448 10.21037/jtd.2017.02.96 PMC5394066

[31] Bradburn MJ , Clark TG , Love SB , Altman DG . Survival analysis part II: multivariate data analysis – an introduction to concepts and methods. Br J Cancer. 2003;89(3):431‐436. 12888808 10.1038/sj.bjc.6601119 PMC2394368

