# Supplementary material for: Assessment of lung cancer risk factors and mortality in Qatar: A case series study
Source: Cancer Rep. Author manuscript; Available in PMC 2021 Jul 30. (PMC7941510; doi:10.1002/cnr2.1302)
Supplement: Supplementary Material [file NIHMS1676019-supplement-Supplementary_Material.docx]

**Supplementary Materials**

1. **Figures**


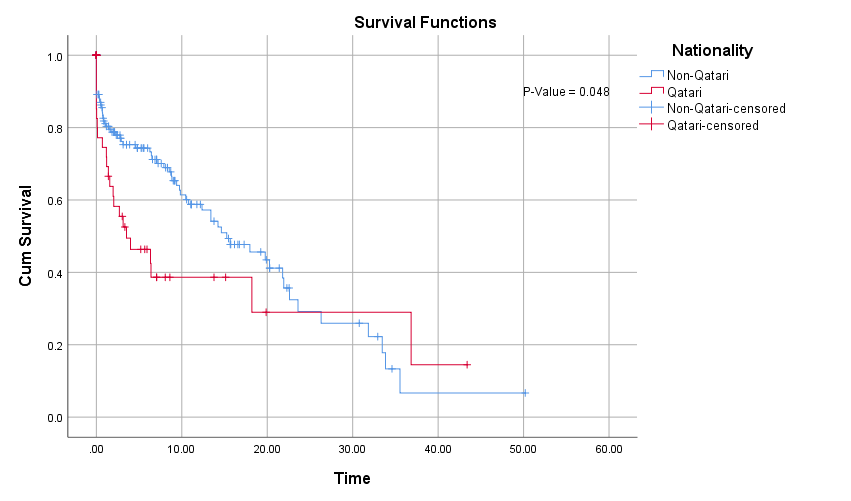


Figure SF1: Kaplan-Meier survival curves for lung cancer patients varying with nationality


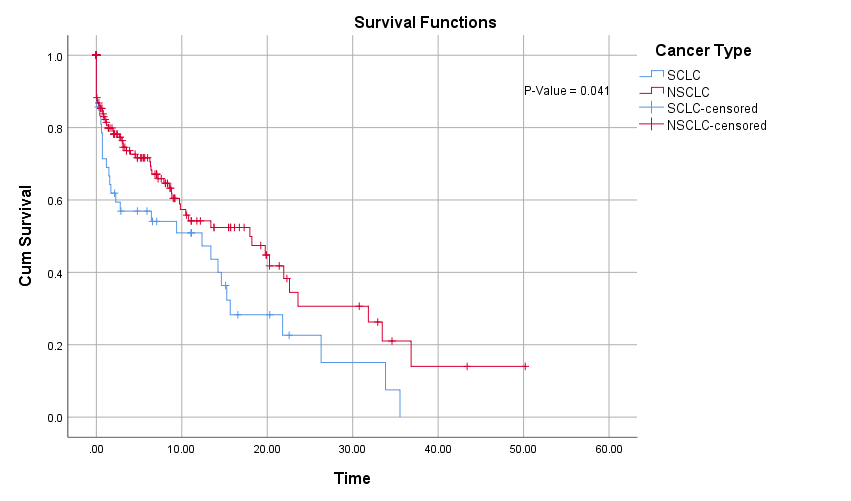


Figure SF2: Kaplan-Meier survival curves for lung cancer patients varying with lung cancer types


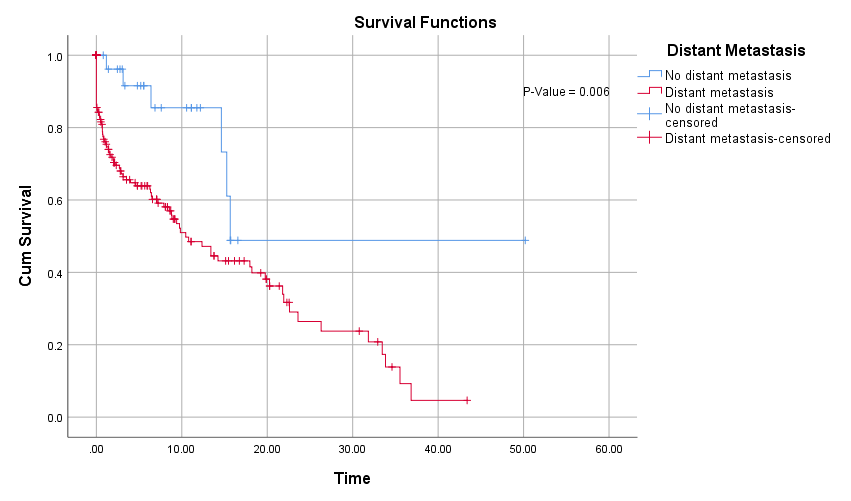


Figure SF3: Kaplan-Meier survival curves for lung cancer patients varying with metastasis


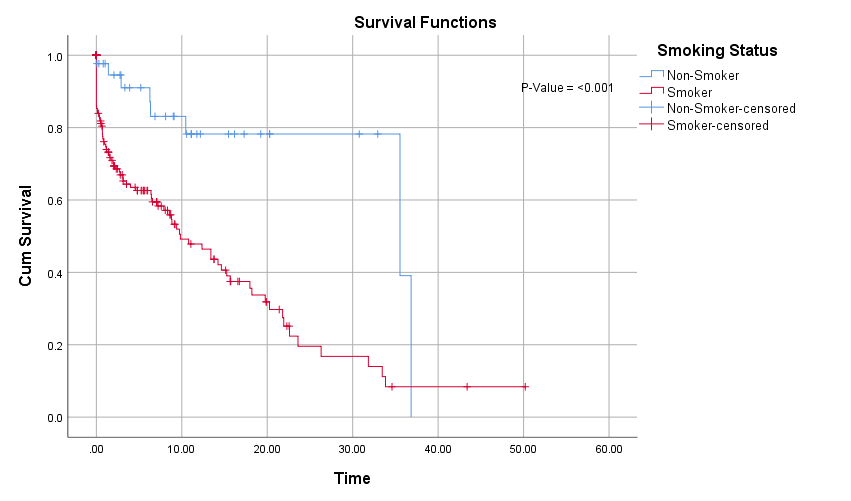


Figure SF4: Kaplan-Meier survival curves for lung cancer patients varying with smoking status at the time of cancer diagnosis


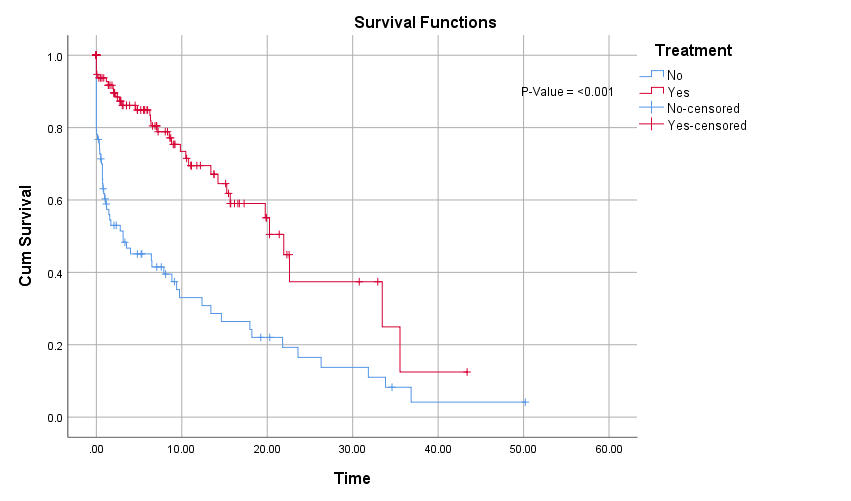


Figure SF5: Kaplan-Meier survival curves for lung cancer patients varying with treatments


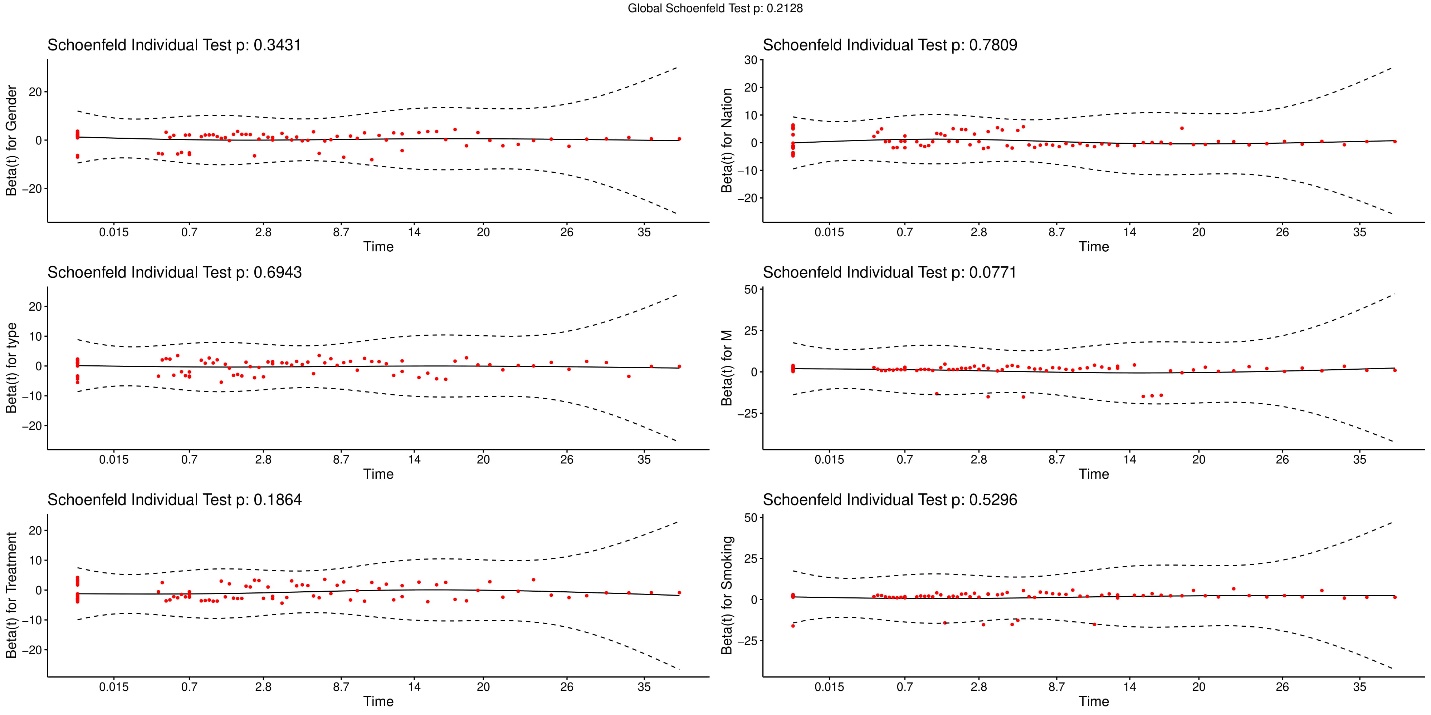


Figure SF6: Scaled Schoenfeld residuals plotted against the transformed time for the various covariates in the Cox model. Corresponding p-values from the chisquare test are also provided.

1. **Tables**

*Table ST1: Patient characteristics across the vital status. The p-values correspond to the chi-square tests.*

|  | **Vital Status** | | |
| --- | --- | --- | --- |
|  | Alive  N (%) | Dead  N (%) | p-value |
| **Gender** |  |  |  |
| Female | 39 (69.6) | 17 (30.4) | 0.044 |
| Male | 94 (54.3) | 79 (45.7) |  |
| **Nationality** |  |  |  |
| Non-Qatari | 105 (60) | 70 (40) | 0.289 |
| Qatari | 28 (51.9) | 26 (48.1) |  |
| **Cancer Type** |  |  |  |
| SCLC | 19 (38) | 31 (62) | 0.001 |
| NSCLC | 114 (63.7) | 65 (36.6) |  |
| **Distant metastasis** |  |  |  |
| No | 27 (81.8) | 6 (18.2) | 0.003 |
| Yes | 106 (54.1) | 90 (45.9) |  |
| **Smoking Status at the time of cancer diagnosis** |  |  |  |
| Non-Smoker | 35 (81.4) | 8 (18.6) | 0.001 |
| Smoker | 98 (52.7) | 88 (47.3) |  |
| **Treatment** |  |  |  |
| No | 36 (37.1) | 61 (62.9) | < 0.001 |
| Yes | 97 (73.5) | 35 (26.5) |  |

*Table ST2: Life table for survival estimates of male lung cancer patients (N=173)*

| Years | Interval (Month) | | Failed | Censored | Survival Rate | SE |
| --- | --- | --- | --- | --- | --- | --- |
|  | Lower | Upper |  |  |  |  |
| 1^st^ | 0 | 12 | 63 | 73 | 1.000 | 0.000 |
| 2^nd^ | 12 | 24 | 11 | 16 | 0.539 | 0.043 |
| 3^rd^ | 24 | 36 | 4 | 3 | 0.334 | 0.055 |
| 4^th^ | 36 | 48 | 1 | 1 | 0.177 | 0.064 |
| 5^th^ | 48 | 60 | 0 | 1 | 0.106 | 0.067 |

*Table ST3: Life table for survival estimates of female lung cancer patients (N=56)*

| Years | Interval (Month) | | Failed | Censored | Survival Rate | SE |
| --- | --- | --- | --- | --- | --- | --- |
|  | Lower | Upper |  |  |  |  |
| 1^st^ | 0 | 12 | 12 | 36 | 1.000 | 0.000 |
| 2^nd^ | 12 | 24 | 4 | 3 | 0.684 | 0.075 |
| 3^rd^ | 24 | 36 | 1 | 0 | 0.263 | 0.134 |
| 4^th^ | 36 | 48 | 0 | 0 | 0.263 | 0.134 |
| 5^th^ | 48 | 60 | 0 | 0 | 0.263 | 0.134 |
